# *Acrocomia aculeata* (Jacq.) Lodd. ex Mart. Leaves Increase SIRT1 Levels and Improve Stress Resistance

**DOI:** 10.1155/2020/5238650

**Published:** 2020-03-22

**Authors:** Tamaeh Monteiro-Alfredo, Paulo Matafome, Bianca Pancoti Iacia, Kátia Ávila Antunes, Jéssica Maurino dos Santos, Janielle da Silva Melo da Cunha, Sara Oliveira, Alex Santos Oliveira, Jaqueline Ferreira Campos, Mariana Magalhães, Célia Cabral, Raquel Seiça, Cláudia Andrea Lima Cardoso, Caio Fernando Ramalho de Oliveira, Edson Lucas dos Santos, Kely de Picoli Souza

**Affiliations:** ^1^Research Group on Biotechnology and Bioprospecting Applied to Metabolism (GEBBAM), Federal University of Grande Dourados, Dourados, MS, Brazil; ^2^Institute of Physiology, Faculty of Medicine, University of Coimbra, Coimbra, Portugal; ^3^Coimbra Institute for Clinical and Biomedical Research (iCBR), Faculty of Medicine; CNC.IBILI Consortium & CIBB Consortium, University of Coimbra, 3000-548 Coimbra, Portugal; ^4^Department of Complementary Sciences, Instituto Politécnico de Coimbra, Coimbra Health School (ESTeSC), Coimbra, Portugal; ^5^Centre for Functional Ecology, Department of Life Sciences, University of Coimbra, Calçada Martim de Freitas, 3000-456 Coimbra, Portugal; ^6^Course of Chemistry, State University of Mato Grosso do Sul, Dourados, 79070-900 Mato Grosso do Sul, Brazil

## Abstract

Oxidative stress is a metabolic disorder linked with several chronic diseases, and this condition can be improved by natural antioxidants. The fruit pulp of the palm *Acrocomia aculeata* (Jacq.) Lodd. ex Mart. is widely used in the treatment of various illnesses, but as far as we know, there are no reports regarding the properties of its leaves. Thus, we aimed to evaluate the antioxidant activity of *A. aculeata* leaf extracts obtained with water (EA-Aa), ethanol (EE-Aa), and methanol (EM-Aa) solvents. The extracts were chemically characterized, and their antioxidant activity was assessed through the scavenging of the free radicals DPPH and ABTS. EE-Aa and EM-Aa showed the highest amounts of phenolic compounds and free radical scavenging activity. However, EA-Aa was more efficient to protect human erythrocytes against AAPH-induced hemolysis and lipid peroxidation. Thus, we further show the antioxidant effect of EA-Aa in preventing AAPH-induced protein oxidation, H_2_O_2_-induced DNA fragmentation, and ROS generation in Cos-7 cells. Increased levels of Sirt1, catalase, and activation of ERK and Nrf2 were observed in Cos-7 treated with EA-Aa. We also verify increased survival in nematodes *C. elegans*, when induced to the oxidative condition by Juglone. Therefore, our results showed a typical chemical composition of plants for all extracts, but the diversity of compounds presented in EA-Aa is involved in the lower toxicity and antioxidant properties provided to the macromolecules tested, proteins, DNA, and lipids. This protective effect also proven in Cos-7 and in *C. elegans* was probably due to the activation of the Sirt1/Nrf2 pathway. Altogether, the low toxicity and the antioxidant properties of EA-Aa showed in all the experimental models support its further use in the treatment of oxidative stress-related diseases.

## 1. Introduction

Rising of life expectancy and lifestyle changes are strictly related to overwhelming incidence of chronic diseases worldwide [1]. The negative economic impacts on health systems resulted from the treatment of these diseases, stimulating the development of new, cheaper, and more effective therapeutic alternatives [2]. A key factor involved in the development and progression of many chronic diseases is the oxidative stress [3], which is caused by endogenous (deregulation in the body's redox balance) [4] and exogenous (environmental agents) factors [5]. At low concentrations, reactive species (RS) are physiologically important and participate in cellular signaling of biochemical and immunological processes [6]. A prooxidant condition of the body's redox balance is characterized by the increased RS production, especially reactive species of oxygen (ROS) and nitrogen (RNS). Under these circumstances, the antioxidant defenses are inefficient, failing to effectively neutralize the RS [7]. In excess, RS react with biomolecules such as carbohydrates, lipids, proteins, and nucleic acids, resulting in cellular dysfunction [8] and the development of chronic diseases such as diabetes [9] and cancer [10].

From the pharmacological point of view, medicinal plants are a relevant source of compounds to treat illnesses [11], in particular those related to oxidative stress. The pharmacological activities observed in several plants are due to the constitution of their secondary metabolites, such as flavonoids, tannins, and mainly phenolic compounds [12]. These antioxidant compounds could act alone or in synergism [13], and the positive relationship between the consumption of vegetables rich in antioxidant compounds and the prevention of diseases related to oxidative stress imbalance stimulates the search for plants that prompt both health maintenance and medicinal purposes [14–16].

Brazil owns an important part of the world's biodiversity [17] and exclusive biomes such as Cerrado [18], serving as an important source of raw material for the production of food and herbal medicines [19, 20]. Among the native plants from Cerrado, *Acrocomia aculeata* (Jacq.) Lodd ex Mart., commonly known as *macaúba* or *bocaiúva* [21], is a palm with economic importance due to the large variety of products derived from its fruits and kernel, which are rich in proteins, vitamins, and oils [22]. In addition to its uses in cooking [22], cosmetic industry [21], and biodiesel production [23], it also has been used for ethnopharmacological purposes. In traditional medicine, *A. aculeata* has been used for the treatment of respiratory diseases, such as laxative and analgesic [24], and in the decrease of serum glucose and cholesterol levels [25]. Several chemical compounds with high antioxidant potential have already been described in *A. aculeata*. The fruit pulp is known to contain oleic acid [26], *β*-carotene [27], and *α*-tocoferol, whereas the kernel has lauric and oleic acids [28]. Although fruits are more widely used and studied, the leaves are used in animal nutrition [29] and no toxicity has been reported [30]. Given the scarcity of information regarding chemical composition, antioxidant properties, and pharmacological potential, we aimed to determine the chemical composition, toxicity, and antioxidant properties of *A. aculeata* leaves with the goal to obtain accurate information about this plant part underutilized.

## 2. Material and Methods

### 2.1. Chemicals and Antibodies

Organic solvents and salts used in all experiments were purchased from Sigma-Aldrich/Merck, Biowest, and Fischer Scientific. Antibodies used were targeted to Sirt1, ERK, phospho-ERK-Thr202/Tyr204 (Cell Signalling), Nrf2 (Santa Cruz Biotechnology), and phospho-Nrf2-Ser40 (Invitrogen). Calnexin was used as the loading control (Sicgen, Portugal).

### 2.2. Plant Material

*A. aculeata* fresh leaves were collected in the morning, between July and August of 2014, in the region of Grande Dourados, Macaúba district, state of Mato Grosso do Sul (MS) (22°07′02.4^″^ S 54°28′36.3^″^ W), under the permission of the Brazilian Biodiversity Authorization and Information System (Sistema de Autorização e Informação sobre Biodiversidade, SISBIO; no. 50589). The species was identified by a plant taxonomist, and a voucher specimen was deposited in the herbarium (DDMS-UFGD) of the Federal University of Grande Dourados, Dourados (MS), Brazil, under the registration number 5103.

Leaves were washed, cut into 5 cm strips, and dried in an air circulation oven at 45 ± 5°C until the mass of the samples was stabilized. The dried leaves were ground in a Willey-knife mill and sieved in a 10 mm mesh, and the resulting powder was stored at -20°C.

### 2.3. Isolation of Extracts

The aqueous extract of *A. aculeata* (EA-Aa) was obtained by infusion; the powder was mixed with boiled water (100 g/L) under manual shaking until its complete cooling. After, the extract was filtered, freeze-dried, and stored at -20°C. The ethanolic (EE-Aa) and methanolic (EM-Aa) extracts were prepared adding the leaf powder in ethanol and methanol, respectively, at the same proportion of EA-Aa. The extraction occurred by maceration during 7 days, followed by the process of concentration in a rotary evaporator and freeze-dried.

### 2.4. Chemical Composition

#### 2.4.1. Total Phenolic Compounds, Flavonoids, and Condensed Tannins

Total phenolic compound of the extracts was determined by spectrophotometry (T70 UV/VIS Spectrometer, PG Instruments Ltd), using Folin-Ciocalteu reagent, and the results were expressed in milligram equivalents of gallic acid per gram of extract (mg EAG^−1^) [31]. Total flavonoid content was determined using a 2% aluminium chloride solution in methanol as the reagent, resulting in an average value expressed in milligram equivalents of quercetin per gram of extract (mg·EQ^−1^) [32]. The amount of condensed tannins was also determined by a spectrophotometric method, using a solution of vanillin in 8% acidified methanol [33]. The results were expressed in milligram equivalents of catechin per gram of extract (mg·EC^−1^). All experiments were carried out in three independent experiments in triplicate.

#### 2.4.2. Chromatographic Analysis by LC-6AD

The EA-Aa, EE-Aa, and EM-Aa were solubilized in water : methanol (8 : 2, *v* : *v*) and evaluated in a LC analytical column (LC-6AD Shimadzu, Kyoto, Japan) with the aid of a photodiode array detector (PDA) system which was monitored between wavelengths *λ* = 200-800 nm, in an LC analytical apparatus, where the column was ODS HYPERSIL (C-18, 150 mm long × 4.6 mm diameter, Thermo Electron Corporation). The flow quotient and the injection volume were, respectively, 1 mL·min^−1^ and 10 *μ*L. All the chromatographic analysis took place at a temperature of 25°C. Eluent A was composed of a binary mobile phase of water with 6% acetic acid and 2 mM of sodium acetate, and eluent B was composed of acetonitrile and the following gradient was applied: 0 min 5% B; 20 min 15% B; 30 min 60% B; and 40 min 100% B. Samples of vanillic acid, caffeic acid, ferulic acid, rosmarinic acid, p-coumaric acid, rutin, quercetin, luteolin, apigenin, and vanillin were used, prepared in methanol-water at a concentration of 1,000 *μ*g·mL^−1^ in 1 : 1 (water : methanol, *v* : *v*). The identification of the compounds with the aid of PDA detector scanning in the spectral range of 200-800 nm did not reveal interferences in retention time of the samples in LC by the developed elution method. Standards were identified and quantified based on their absorption spectra in the UV region and in retention time. Standards found in extracts were unambiguously identified by performing coinjection experiments in which aliquots of the extracts, and standards were mixed and diluted to a known volume and analyzed through LC. The calibration curves were determined by linear regression using LC. The linearity for standards was assessed for 10 concentration ranges. The average standard errors for the peak areas of replicated injections (*n* = 5) were less than 2%, thus showing good repeatability of the calibration curve. The respective coefficients of determination (*r*^2^) were 0.9994 for caffeic acid, vanillic acid, ferulic acid, and gallic acid, and *r*^2^ is 0.9996 for rutin and quercetin.

#### 2.4.3. Chromatographic Analysis by GC-MS

To prepare samples for CG-MS analysis, 2 mL of water and 2 mL of hexane were added separately to 100 mg of each *A. aculeata* extract (EA-Aa, EE-Aa, and EM-Aa); after phase formation, the hexane fraction was separated from the aqueous fraction. To the aqueous fraction, 2 mL of hexane was added and the process was repeated. After the two extractions, the hexane fractions were dried and suspended in 1,000 mL hexane. For GC-MS analysis, the solution was first filtered through a 0.45 *μ*m ultrafilter.

To identify the compounds present in EA-Aa, EE-Aa, and EM-Aa, the samples were also evaluated by mass spectrometry (GC-MS). The GC-MS analysis was performed using a GC-2010 Plus, Shimadzu, Kyoto, Japan, equipped with a mass spectrometry detector (GC-MS Ultra 2010), using LM-5 (5% phenyl dimethylpolysiloxane), capillary column of fused silica (15 m length × 0.2 mm diameter and 0.2 *μ*m thick film). The analysis took place under the following conditions: helium entrainment gas (99.999% and flow rate 1 mL·min^−1^), 1 *μ*L of injection volume, division ratio (1 : 20), furnace initial temperature adjusted to 150°C, and heating at 150°C to 280°C at 15°C·min ^−1^ and a hold at 280°C for 15 min. The injector temperature was 280°C, and the quadrupole detector temperature was 280°C. The MS scanning parameters included an electron impact ionization voltage. The identifications were performed by comparing the mass spectra obtained in the NIST21 and WILEY229 libraries. In some cases, when the identified spectra were not found, only the structural type of the corresponding component was proposed based on its mass spectral fragmentation. When possible, reference compounds were cochromatographed to confirm GC-retention times. Standards of the stigmasterol, campesterol, *β*-sitosterol, lupeol, and lupeol acetate were prepared in hexane in the concentration of 1,000 *μ*g·mL^−1^. The concentrations of compounds were determined by extern calibration. The linearity for standards was assessed for 5 concentration ranges. The average standard errors for the peak areas of replicated injections (*n* = 5) were less than 2%, thus showing good repeatability of the calibration curve. The respective coefficients of determination (*r*^2^) were 0.9996 for stigmasterol, campesterol, *β*-sitosterol, and lupeol, and *r*^2^ is 0.9994 for lupeol acetate.

### 2.5. Free Radical Scavenging Potential

#### 2.5.1. 2,2-Diphenyl-1-picrylhydrazyl (DPPH)

The antioxidant activity of the EA-Aa, EE-Aa, and EM-Aa was evaluated through the capture of the free radical 2,2-diphenyl-1-picrylhydrazyl (DPPH). DPPH control solutions (0.11 mM) were incubated with positive control, ascorbic acid (AA), and extracts, prepared in different concentrations (0.1–2,000 *μ*g·mL^−1^) during 30 min, at room temperature, protected from light. After this period, spectrophotometer readings were performed at 517 nm. To calculate the percentage of inhibition of the free radical, the absorbance values of the samples and controls according to equation (1) were used [34]. Three independent experiments were performed in triplicate.

(1) $$\begin{array}{c} \%\text{DPPH inhibition}=\left.\left(\frac{{\text{Abs}}_{\text{control DPPH}}-{\text{Abs}}_{\text{sample}}}{{\text{Abs}}_{\text{control DPPH}}\;}\right.\right)\times 100. \end{array}$$

#### 2.5.2. 2,2′-Azino-bis(3-ethylbenzothiazoline-6-sulphonic Acid) (ABTS)

The scavenging capacity of the extracts was also determined with the free radical 2,2′-azino-bis(3-ethylbenzothiazoline-6-sulphonic acid) (ABTS). The stock solution of ABTS was previously prepared (12 to 16 h) from an oxidation reaction between potassium persulfate (2.45 mM) and ABTS (7 mM). The analysis was performed at the same concentrations as the DPPH assay; the spectrophotometer readings occurred at 734 nm after 6 min of reaction among the solution of ABTS and the extracts. The results were expressed as % of free radical scavenging, according to equation (2). Three independent experiments were performed [35].

(2) $$\begin{array}{c} \%\text{ABTS inhibition}=\left.\left(\frac{{\text{Abs}}_{\text{control ABTS}}-{\text{Abs}}_{\text{sample}}}{{\text{Abs}}_{\text{control ABTS}}\;}\right.\right)\times 100. \end{array}$$

### 2.6. Oxidative Hemolysis Assay

#### 2.6.1. Toxicity in Erythrocytes

In order to evaluate the hemolytic effect of the extracts, peripheral whole blood of a single adult healthy donor was collected and stored in tubes containing anticoagulant sodium citrate. Experiments were performed under the approval of the Research Ethics Committee CEP/UFGD no. 5160. Then, a solution of 10% of erythrocytes in physiological solution (NaCl 0.9%) was prepared. Afterwards, erythrocytes were incubated with the positive control, ascorbic acid (AA), and the extracts EA-Aa, EE-Aa and EM-Aa in different concentrations (25–1,000 *μ*g·mL^−1^) at 37°C during 4 h, under constant shaking. In sequence, samples were centrifuged and the supernatant was read at 540 nm. Results were expressed as the percentage of hemolysis. Three independent experiments were performed in duplicate [36].

#### 2.6.2. Oxidative Hemolysis Assay Induced by 2,2′-Azobis(2-amidinopropane) Dihydrochloride (AAPH)

Following the evaluation of extract toxicity in erythrocytes, the protective effect against oxidative hemolysis was investigated. Hence, the same conditions and concentrations of the prior assay were repeated and 50 mM of 2,2′-azobis(2-amidinopropane) dihydrochloride (AAPH) was used to induce lipid peroxidation of erythrocytes membrane. Hemolysis was determined as the previous assay [36].

#### 2.6.3. Quantification of Malondialdehyde (MDA) Generation

After the incubation of the erythrocytes with AAPH, as described above, an aliquot of the supernatant was added to 20 nM of thiobarbituric acid (TBA) and incubated at 96°C during 45 min. After, the tubes were placed in an ice bath during 15 min to stop the reaction and 4 mL of butanol was added to the tubes, with subsequent vortex and centrifugation at 3,000 rpm during 5 min. The absorbance of the supernatant was performed in a spectrophotometer at 532 nm. The generation of the lipid peroxidation product, MDA, was expressed by the following [36]:

(3) $$\begin{array}{c} \text{MDA nmol}\cdot {\text{mL}}^{-1}=\frac{{\text{Abs}}_{\text{sample}}\left(20\times 220.32\right)}{{\text{Abs}}_{\text{standard MDA}}\;}. \end{array}$$

### 2.7. Protein Oxidation

The protective effect of extracts on protein oxidation was investigated using bovine serum albumin (BSA) as the standard. An amount of 3 *μ*g of BSA was preincubated with 3 *μ*L of EA-Aa in different concentrations (25–1,000 *μ*g·mL^−1^) during 30 min. Afterwards, 3 *μ*L of 120 mM AAPH was added to each tube and further incubated at 37°C during 1 h. At the end of incubation, the samples were mixed with sample buffer, heated at 95°C during 3 min and applied onto 12% SDS-PAGE. The gels were solved in Mini-PROTEAN Tetra Cell (Bio-Rad Laboratories, CA, USA) at 200 V during 60 min and digitalized in Gel Doc EZ Imager (Bio-Rad Laboratories). The band volume was determined with the Image Lab software. The increase in band volume was considered a protein oxidative damage [37]. Afterwards, the difference between the BSA standard band volume and the remaining bands was obtained and graphed, displaying the protection against the protein oxidative damage according with different concentrations of EA-Aa.

### 2.8. DNA Fragmentation

The DNA damage was performed through the induction with H_2_O_2_. For this, plasmid DNA (50 ng·*μ*L^−1^) in a PBS solution was incubated with EA-Aa (25–1,000 *μ*g·mL^−1^) or standard controls, rutin (R), quercetin (Q), gallic acid (A), or catechin (C) (125 *μ*g·mL^−1^), and H_2_O_2_ 30%. Samples were incubated in a transilluminator UVT-312 at 302 nm, at room temperature during 5 min, followed by loading and electrophoresis in agarose gel 2% with ethidium bromide (10 mg·mL^−1^). Gel was scanned by a Gel Doc™ EZ System photodocumenter, and the analysis was performed with Image Lab™ software. Experiments were realized in triplicate [38].

### 2.9. *In Vitro* Antioxidant Activity in Cos-7

#### 2.9.1. Cell Culture

Fibroblast cell line derived from a green monkey (*Cercopithecus aethiops*) kidney (Cos-7) was cultured in Dulbecco's modified Eagle medium-high glucose (DMEM-HG), supplemented with 10% fetal bovine serum (FBS) and 1% penicillin/streptomycin, at 37°C and 5% CO_2_. For the experiments, cells were used with an 80% of confluence [39].

#### 2.9.2. Assessment of Cell Viability

To evaluate cell viability, 8 × 10^4^ Cos-7 cells were seeded in 48-well plates. After 24 h, cells were incubated with different concentrations (25–1,000 *μ*g·mL^−1^) of EA-Aa during 24 h, and cell viability was determined through the Alamar blue assay. Briefly, the medium was replaced by a solution of DMEM-HG with 10% of resazurin (0.1 mg·mL^−1^). After 4 h of incubation, the absorbance was measured at 570 nm and 600 nm in a BioTek microplate reader (BioTek Instruments, Inc., Winooski, VT, USA). The data obtained by the Gen5 program were used to calculate Cos-7 viability, according to the following equation [40]:

(4) $$\begin{array}{c} \text{Cos}-7\text{ viability}=\left(\frac{\left({\text{Abs}}_{570}-{\text{Abs}}_{600}\right)\text{of treated cells}}{\left({\text{Abs}}_{570}-{\text{Abs}}_{600}\right)\text{of control cells}}\right)\times 100. \end{array}$$

#### 2.9.3. Antioxidant Activity

To determine the antioxidant activity of the EA-Aa in Cos-7 cells, 8.10^4^ cells were seeded in 96-well plates. After 24 h, cells were incubated with EA-Aa in the same concentrations as the previous assay during 30 min. After this period, the previously determined IC_50_ of the oxidative stress inductor H_2_O_2_ (0.5 *μ*M) was added (data not shown). Following an incubation of 2 h, cell viability was determined according to equation (4).

#### 2.9.4. Fluorescence Immunocytochemistry

In order to assess the role of the EA-Aa in the reduction of ROS formation, 8 × 10^4^ Cos-7 cells were seeded in MilliCells® EZ Slide 8-well glass (Millipore, MA). After 24 h, cells were treated with EA-Aa and H_2_O_2_ in the same conditions used in the previous antioxidant assay. The evaluation of intracellular ROS was realized with 2′,7′-dichlorodihydrofluorescein diacetate (H_2_DCFDA) and with dihydroethidium (DHE) probes, following the manufacturer's instructions (*n* = 3). DAPI was used to stain cell nucleus. Images were obtained with a fluorescence microscope (Zeiss Axio Observer Z1) with an incorporated camera (Zeiss, Germany), detected with 504 nm of excitation and 525 nm of emission for DCF, 587 nm of excitation and 610 nm of emission for DHE, and 353 nm of excitation and 465 nm of emission for DAPI. The settings were kept constant for all analysis. The entire image was used for quantification, and the analysis was performed with ImageJ software.

### 2.10. Western Blot

For Western blot analysis, 1 × 10^6^ Cos-7 cells were seeded in 6-well plates during 24 h and then treated with EA-Aa (500–1,000 *μ*g·mL^−1^) for 24 h. After, cells were washed with ice-cold PBS, disrupted in lysis buffer (0.25 M Tris-HCl, 125 mM NaCl, 1% Triton-X-100, 0.5% SDS, 1 mM EDTA, 1 mM EGTA, 20 mM NaF, 2 mM Na_3_VO_4_, 10 mM *β*-glycerophosphate, 2.5 mM sodium pyrophosphate, 10 mM PMSF, and 40 *μ*L of protease inhibitor), subjected to three freeze/thaw cycles in liquid nitrogen, and centrifuged (14,000 rpm, 20 min, 4°C). Protein concentration of the supernatant was measured through the BCA Protein Assay Kit [41], and the samples were mixed with Laemmli buffer (62.5 mM Tris-HCl, 10% glycerol, 2% SDS, 5% *β*-mercaptoethanol, 0.01% bromophenol blue). An amount of 15 *μ*g of total protein was loaded onto 8% SDS-PAGE, solved and electroblotted onto PVDF membrane. A rainbow marker was used as a standard weight protein marker. Membranes were blocked with TBS-T 0.01% and 5% BSA and incubated with primary antibody (against Sirt1, ERK, phospho-ERK [Thr202/Tyr204], Nrf2, phospho-Nrf2 [Ser40], and catalase) overnight and with secondary antibodies (anti-rabbit, anti-mouse, and anti-goat) for 2 h at room temperature. Calnexin was used as the loading control. Immunoblots were detected with an ECL substrate and the Versadoc system (Bio-Rad Laboratories, USA).

### 2.11. *In Vivo* Antioxidant Activity in *Caenorhabditis elegans*

The wild-type N2 strain of the nematode *C. elegans*, donated by the Laboratory of Integrative Physiology of the Federal University of São Paulo, was used to access the role of EA-Aa in preventing oxidative damage *in vivo*. Nematodes were cultivated under standard conditions of 15°C and 20°C, in Petri dishes containing Nematode Growth Medium (NGM) agar with a coverage of *E. coli* strain OP50 [42]. To perform the antioxidant experiments, the nematode culture was synchronized through pregnant hermaphrodites and eggs already in plate with 2% sodium hypochlorite and 5 M sodium hydroxide [43].

#### 2.11.1. Evaluation of Nematode Survival

The acute toxicity of the EA-Aa (100–2,500 *μ*g·mL^−1^) was carried out in L4 synchronized worms (*n* = 10 per group), transferred to previously filled 96-well plates with the respective doses of the extracts and M9 buffer. The survival rate was defined after 24 h of incubation at 20°C, with repeatedly physical stimuli with a platinum microspatula, under a stereomicroscope (Motic SMZ-140 and W10X/23; British Columbia, Canada). Three independent experiments were performed in duplicate [44].

#### 2.11.2. Oxidative Stress Assay Induced with 5-Hydroxy-1,4-naphthoquinone (Juglone)

The evaluation of the protective effect of EA-Aa against oxidative stress *in vivo* was performed in *C. elegans* treated with the EA-Aa and exposed to the inducer of intracellular ROS, Juglone. For this, the animals were synchronized (as described in the previous test) and developed in the presence/absence of EA-Aa (500–1,000 *μ*g·mL^−1^). At the L4 stage, animals were transferred to 96-well plates containing M9 buffer and the extract or not. The animals were induced to oxidative stress with the addition of Juglone in an acute and lethal dose, defined in a dose-response curve (data not shown) of 250 *μ*M at 20°C, and the survival of each group was analyzed hourly [44].

### 2.12. Statistical Analysis

The results were expressed as the mean ± standard error of the mean (SEM). We used the Kolmogorov-Smirnov test to determine normality. The analysis of variance (ANOVA) or the Kruskal-Wallis test was used to access differences between groups, and *p* < 0.05 was considered significant.

## 3. Results

### 3.1. Chemical Composition

**Figure 1:**
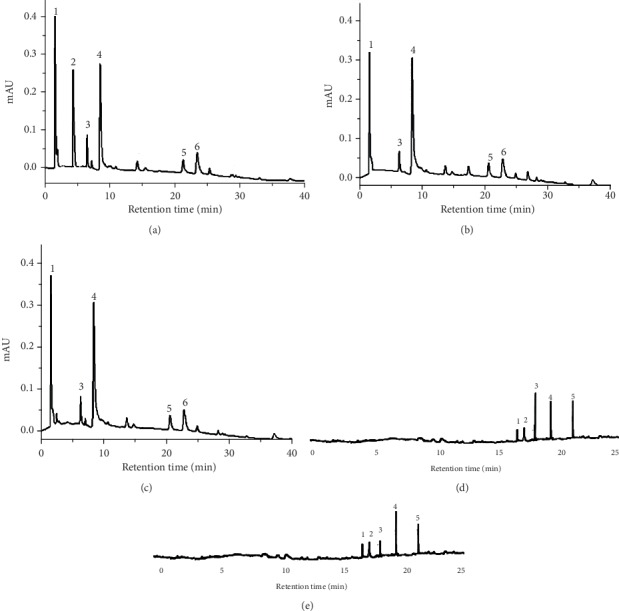
Chromatography analysis of *A. aculeata* leaves by LC-6AD and GC-MS. (a) EA-Aa, (b) EE-Aa, (c) EM-Aa by LC-6AD, (d) EE-Aa, and (e) EM-Aa analysis by GC-MS.

The phytochemical screening of the extracts showed that the alcoholic extractions were responsible for a larger amount of phenolic compounds, flavonoids, and tannins in relation to aqueous extraction. The content of phenolic compounds in EA-Aa, EE-Aa, and EM-Aa was 42.7 ± 2.2, 166.5 ± 1.1, and 175.2 ± 1.8 mg GAE·g^−1^, respectively, the flavonoid content was 16.6 ± 3.2, 78.5 ± 10.8, and 104.5 ± 7.5 mg EG·G^−1^, respectively, and the tannin content was 0.88 ± 0.05, 25.89 ± 1.68, and 18.17 ± 0.53 mg EC·g^−1^, respectively. The chromatographic analysis performed with the extracts by LC-PDA has shown the same peaks (1–6) in all extracts, although peak 2 was only observable in the EA-Aa (Figures 1(a)–1(c)). According to the standards used, reported peaks are gallic acid, vanillic acid, caffeic acid, ferulic acid, rutin, and quercetin. With the exception of vanillic acid, identified only in EA-Aa, a similar content of compounds was shared among the extracts (Table 1). The GC-MS analysis was also performed in the EA-Aa, EE-Aa, and EM-Aa, and the following compounds were identified in EE-Aa and EM-Aa: campesterol, stigmasterol, *β*-sitosterol, lupeol, and lupeol acetate, as shown in Table 2. In the EA-Aa, no compounds were identified.

### 3.2. Antioxidant Activity and Improvement of Oxidative Condition

#### 3.2.1. DPPH and ABTS Free Radical Scavenging

The free radical scavenging potential was assessed using two different free radicals, DPPH and ABTS. The AA was used as a natural and hydrophilic antioxidant positive control. As shown in Table 3, the IC_50_ and maximum activity concentration were lower in EE-Aa and EM-Aa, when compared to EA-Aa, but higher than the AA in both tests, suggesting higher direct free radical scavenging activity of EE-Aa and EM-Aa than EA-Aa.

#### 3.2.2. EA-Aa Reduces Oxidative Stress in Different Biomolecule Sources

**Figure 2:**
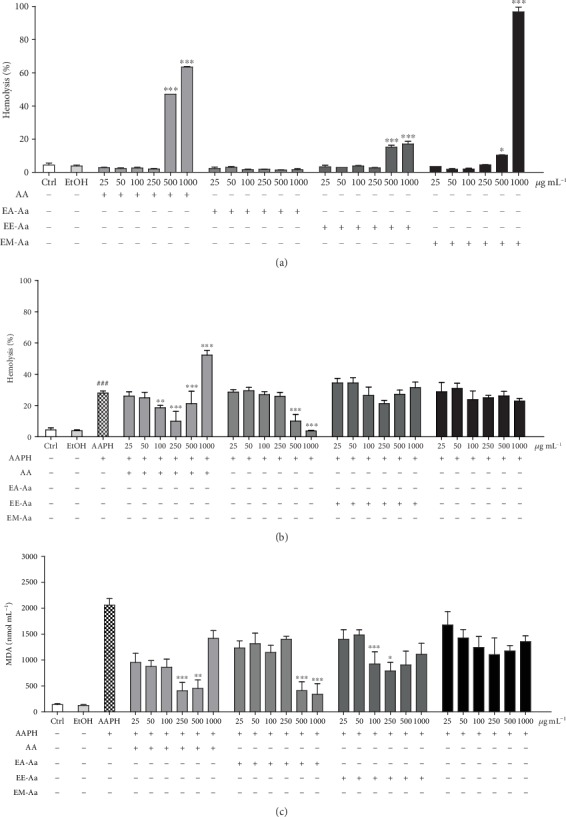
Protective effect of *A. aculeata* extracts in human erythrocytes. (a) Hemolytic effect in human erythrocytes incubated with Aa extracts after 240 min of incubation; no hemolytic effect was observed in EA-Aa, while higher concentrations (500 –1,000 *μ*g·mL^−1^) of the other *Aa* extracts and the positive control AA showed hemolysis at the higher concentrations. (b) Antihemolytic effect of erythrocytes incubated with *Aa* extracts and AAPH (50 mM) after 240 min shows a protective effect of EA-Aa (500–1,000 *μ*g·mL^−1^) against AAPH-induced hemolysis. (c) MDA produced from AAPH-induced lipid peroxidation shows higher protection against lipid peroxidation in the highest concentrations (500–1,000 *μ*g·mL^−1^) of EA-Aa. ^∗^*vs.* Ctrl in (a); ^#^*vs.* Ctrl and ^∗^*vs.* AAPH in (b) and (c); ^∗^*p* < 0.05; ^∗∗^*p* < 0.01; ^∗∗∗,###^*p* < 0.001.

In order to evaluate the role of the *A. aculeata* extracts in a lipid source, human erythrocytes were incubated with growing concentrations of the extracts and the positive control AA. The hemolytic effect of EA-Aa, EE-Aa, and EM-Aa was evaluated, showing hemolysis in the higher concentrations of EE-Aa and EM-Aa, as well as observed for AA (Figure 2(a)). Then, the protective effect of the extracts against AAPH-induced hemolysis was investigated.  Figure 2(b) shows that after 240 min of incubation, the EA-Aa promoted relevant protection with the improvement of AAPH-induced hemolysis. A reduction of 63.84% and 86.60% in relation to the group treated with AAPH was observed in the concentrations of 500 and 1,000 *μ*g·mL^−1^ (Figure 2(b)). Both EE-Aa and EM-Aa did not promote significant protection. In order to evaluate lipid peroxidation, the generation of MDA was determined after the induction with AAPH. In accordance with the hemolysis assay, lower MDA levels were observed in the group treated with EA-Aa (Figure 2(c)). The higher concentrations of EA-Aa (500 and 1,000 *μ*g·mL^−1^) reduced MDA levels in 83.44% and 90.87%, respectively, similarly to the 250 and 500 *μ*g·mL^−1^ of AA, which resulted in a reduction of 84.99% and 78.77% in relation to AAPH. No significant reduction of MDA formation was observed for the EM-Aa, but EE-Aa reduced 55.10% and 61.68% at 100 and 250 *μ*g·mL^−1^, respectively (Figure 2(c)).

**Figure 3:**
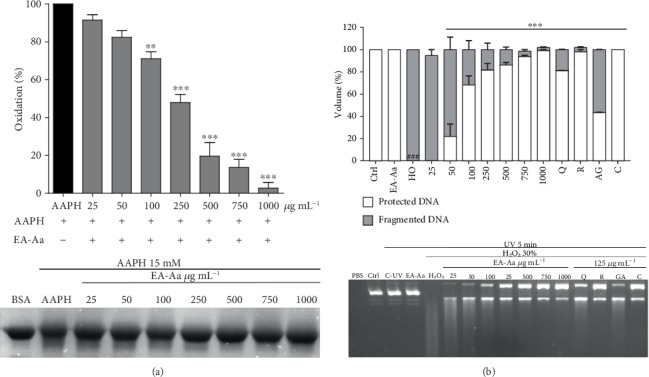
Antioxidant potential of EA-Aa in biomolecules. (a) Protein oxidation induced with AAPH (120 mM) shows a reduction of oxidation with EA-Aa (750–1,000 *μ*g·mL^−1^). (b) Plasmid DNA fragmentation induced with H_2_O_2_ (30%) showing protection by EA (50 *μ*g·mL^−1^–1,000 *μ*g·mL^−1^) and positive controls, quercetin, rutin, gallic acid, and catechin. ^#^*vs.* Ctrl; ^∗^*vs.* AAPH/H_2_O_2_; ^∗∗^*p* < 0.01; ^###,∗∗∗^*p* < 0.001.

Since EA-Aa promoted the highest protection against oxidative hemolysis and MDA formation, this extract was selected to further antioxidant activity assays. Therefore, the role of EA-Aa in protein protection against oxidative damage induced from AAPH and DNA protection against UV-induced damage were assessed.  Figure 3(a) shows the antioxidant effect of EA-Aa against AAPH-induced protein oxidation, demonstrating a clear concentration-dependent protection, with a decrease of fuzzy bands with higher EA-Aa concentrations. Similar results were observed in the DNA fragmentation assay, shown in Figure 3(b). A complete loss of DNA integrity was observed following exposure to UV and H_2_O_2_. Increasing concentrations of EA-Aa promoted a complete protection of plasmidial DNA, similar to the standard controls quercetin, rutin, gallic acid, and catechin (Figure 3(b)).

### 3.3. EA-Aa Reduces *In Vitro* and *In Vivo* Oxidative Stress and Improves Both Cell Viability and Nematode Survival

**Figure 4:**
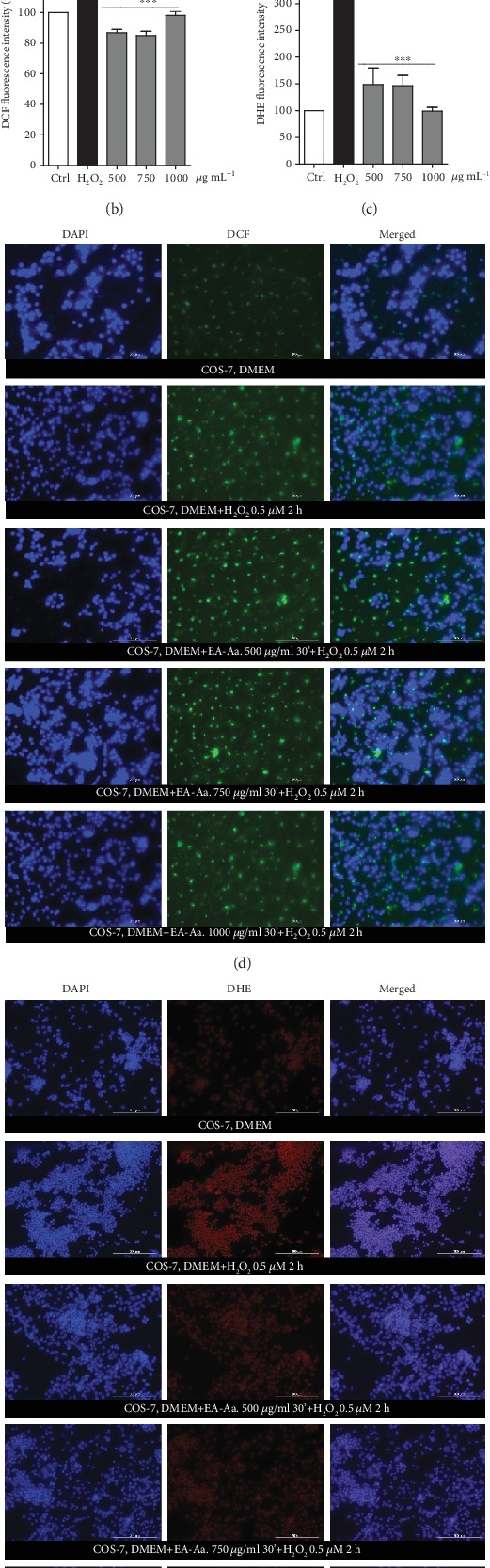
Inhibition of ROS generation by EA-Aa in Cos-7 cells. (a) Viability of Cos-7 cells treated with EA-Aa (500-1,000 *μ*g·mL^−1^) incubated with or without H_2_O_2_ (0.5 *μ*M), shows low cytotoxicity of EA-Aa and protection (750–1,000 *μ*g·mL^−1^) against H_2_O_2_-induced oxidative damage from the decrease of H_2_O_2_-induced ROS (stained with DCF) (b) and O_2_^·-^ generation (stained with DHE) (c) with EA-Aa (500–1,000 *μ*g·mL^−1^). Representative images of DCF and DHE are shown in (d) and (e), respectively. ^#^*vs.* control; ^∗^*vs.* H_2_O_2_; ^#^*p* < 0.05; ^##^*p* < 0.01; ^###,∗∗∗^*p* < 0.001.

**Figure 5:**
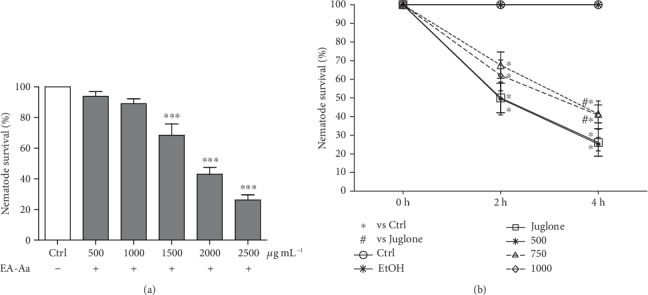
In vivo antioxidant potential of EA-Aa in *C. elegans*. (a) EA-Aa shows toxicity in the nematodes only in concentrations higher than 1,500  *μ*g·mL^−1^. (b) Antioxidant potential of EA-Aa (500-1,000 *μ*g·mL^−1^) prevented Juglone-induced lower *C. elegans* survival after 4 h. ^∗^*vs.* Ctrl; ^#^*vs. Juglone;*^∗,#^*p* < 0.05; ^∗∗∗^*p* < 0.01.

The antioxidant and cytoprotective effects of EA-Aa were further confirmed in higher complexity models, namely, *in vitro* in the cell line Cos-7 and *in vivo* in the nematode *C. elegans*. The IC_50_ of the inducers, H_2_O_2_ (Cos-7) and Juglone (*C. elegans*), were determined (data not shown). The EA-Aa-induced cytotoxicity was determined after incubation with growing concentrations during 24 h. A slight reduction of Cos-7 viability was observed with increasing concentrations of EA-Aa (Figure 4(a)). Up to 1,000 *μ*g·mL^−1^, no effects on *C. elegans* survival were noticed. To affect *C. elegans* survival was necessary to increase the EA-Aa concentration in 50, 100, and 150% (Figure 5(a)). In order to determine the protective role of EA-Aa *in vitro*, Cos-7 cells were incubated with 0.5 *μ*M H_2_O_2_ (IC_50_). At higher concentrations, EA-Aa was able to rescue Cos-7 cells treated with H_2_O_2_, displaying an increase of 26.06% (750 *μ*g·mL^−1^) and 33.08% (1,000 *μ*g·mL^−1^) in cell viability in relation to H_2_O_2_-treated cells (Figure 4(a)). *C. elegans* survival was performed after the induction of oxidative stress with 250 *μ*M Juglone (IC_50_). EA-Aa led to an increase of 17.58% and 12.12% of nematode survival in the concentrations of 750 *μ*g·mL^−1^ and 1,000 *μ*g·mL^−1^ after 2 h of incubation. Following 4 h of Juglone exposure, EA-Aa increased the worm survival in 15.32% (750 *μ*g·mL^−1^) and 14.65% (1,000 *μ*g·mL^−1^).

In order to evaluate the protection against oxidative stress generation in Cos-7 cells, probes to total ROS (DCF) and to superoxide anion (DHE) were used. Increased staining to DCF (Figures 4(b)and 4(d)) and DHE (Figures 4(c) and 4(e)) was observed following the incubation of the cells with 0.5 *μ*M H_2_O_2_ in relation to control cells.

The incubation of EA-Aa at the concentrations that protected cells against oxidative damage induced by H_2_O_2_, (750 and 1,000 *μ*g·mL^−1^) reduced the fluorescence signal from both probes at levels observed in control cells, showing that EA-Ae was able to neutralize the formation of ROS and superoxide anion, corroborating the previous assay of cellular viability where these concentrations increased cellular viability.

### 3.4. EA-Aa Activates Sirt1, ERK, and Nrf2 Pathway

**Figure 6:**
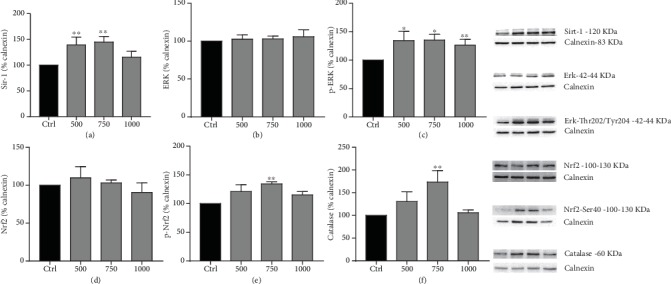
Mechanisms involved in EA-Aa-mediated protection of Cos-7 cells. Western blot analysis shows that EA-Aa increased the levels of Sirt-1 (a), ERK-Thr202/Tyr204 (c), Nrf2-Ser40 (e), and catalase (f), mostly at 750 *μ*g·mL^−1^. No alterations were observed in the total levels of ERK (b) and Nrf2 (d). ^∗^*vs.* Ctrl, ^∗^*p* < 0.05; ^∗∗^*p* < 0.01.

The pathways activated by the EA-Aa were further assessed in Cos-7 cells by Western blotting analysis. Following the incubation of EA-Aa during 24 h, Sirt1 levels increased in 500 *μ*g·mL^−1^ and 750 *μ*g·mL^−1^ (Figure 6(a)). While increased phosphorylated ERK (p-ERK) (Thr202/Tyr204) was observed in all concentrations, phosphorylated Nrf2 (p-Nrf2) (Ser40) and catalase levels were observed only in the group treated with 750 *μ*g·mL^−1^of EA-Aa. No changes were observed in ERK and NRF2 levels (Figures 6(a)–6(f)).

## 4. Discussion

About 70% of the worldwide population consumes medicinal plants as a therapeutic purpose [45]. In the last decades, medicinal plants have been the focus of many studies on the investigation of therapeutic potentials, which are largely related to their secondary metabolites, such as phenolic compounds [46, 47]. Accordingly, this study characterizes the chemical composition of *A. aculeata* leaves, through the quantitative determination of phenolic compounds by colorimetric and chromatographic methods, as well as the determination of its antioxidant properties and the involved mechanisms.

Phenolic compounds are a class of plant secondary metabolites that act by redox chelation of metal ions, inhibiting the conversion of hydroperoxides into a RS and as a free radical scavenger by donating electrons from its hydroxyl groups [48]. Among this class, flavonoids are associated with several pharmacological benefits, widely distributed in plants, with important antioxidant activity contribution [49], already described in *A. aculeata* fruit pulp [24] and in other palm trees [50, 51]. Its potential comes from the donation of hydrogens or electrons to promote the stabilization of intermediate radical, also acting as an iron chelating agent [52].

The phenolic compounds identified in *A. aculeata* extracts were gallic, caffeic, vanillic, and ferulic acids and the flavonoids rutin and quercetin, which are likely to be linked to the potential of *A. aculeata* extracts in the antioxidant assays, as already reported in some studies with other plants [36, 48, 53–55]. GC-MS analysis also identified campesterol, stigmasterol, and *β*-sitosterol, as well as the triterpenes lupeol and lupeol acetate, which are known to possess antioxidant properties [56] and anti-inflammatory effects [57]. These molecules have been investigated with pharmacological purposes in cancer, diabetes, and cardiovascular diseases [58]. Triterpenes were only identified in EE-Aa and EM-Aa; its presence might be related to lower IC_50_ values obtained in the free radical scavenging tests of DPPH and ABTS in comparison with EA-Aa.

The protective properties of *A. aculeata* extracts on oxidative stress-induced damage were assessed in lipid, protein, and DNA. Isolated erythrocytes were used to assess lipid peroxidation resulting from AAPH-mediated attack on cell membranes and ROS formation. This compound generates hydroxyl radicals [59] and a lipid peroxide radical (LOOH) after chain reactions in the polyunsaturated fatty acids (PUFAs) from cell membranes. As result of the oxidative chain reaction, *α*- and *β*-aldehydes are formed, such as MDA, 4-hydroxynonenal (4-HNE), and acrolein, as well as isoprostanes [60], which are collectively named as thiobarbituric reactive species (TBARS) [61]. The accumulation of TBARS could lead to cell dysfunction because of the damage of its structure, culminating in cell death [62]. In fact, to check the protective effect in *A. aculeata* extracts, we analyzed the AAPH-induced oxidative hemolysis and MDA generation. In this way, the protection against oxidation is considered beneficial for the maintenance of health and prevention against the development of several diseases [53]. Similar results were also obtained in Cerrado plants [36, 63]. The prooxidant effect showed by EE-Aa and EM-Aa in high concentrations is probably caused by the generation of free radicals, same as reported with AA [64]. The distinct chemical composition of EA-Aa allowed no toxic outcome in the same concentrations of the other two extracts and promoted antioxidant protection at higher concentrations; thus, it was chosen for further experiments.

The protective effects of the EA-Aa were further evaluated *in vitro* and *in vivo* in Cos-7 cells and nematodes *C. elegans*, respectively. The EA-Aa was considered safe due to its low toxicity (cell viability higher than 80%) even at high concentrations (1,000 *μ*g·mL^−1^). In Cos-7, the extract rescued cell viability in the presence of H_2_O_2_ at 750 *μ*g·mL^−1^ and 1,000 *μ*g·mL^−1^, and we also proved that EA-Aa were able to improve H_2_O_2_-induced ROS formation, assessed by the fluorescent probes H_2_DCFDA and DHE [65]. This kind of oxidative protection is crucial in the protection against a lot of diseases related to this condition [3].

Although *in vitro* models are safe, like we used Cos-7 cells, they are the same set of cells that have the same function, and the effect of the extract could be underestimated, so we decided to evaluate the potential of the extract in *in vivo* models as well. And with regard to the protection against ROS generation, the effects of EA-Aa were reproducible in *C. elegans*, where the oxidative damage is induced by Juglone, an intracellular ROS generator that decreases viability and survival. So, the nematode survival was improved, by the prevention of Juglone-induced oxidative damage [66]. *A. aculeata* fruit pulp and kernel have shown similar results, in particular the *in vitro* antioxidant activity and absence of cytotoxicity [67], besides hypoglycemic [68] and anti-inflammatory and diuretic effects [69].

In order to understand the mechanisms involved in EA-Aa-induced prevention of oxidative damage, the levels of key enzymes involved in the response to cell stress were evaluated. EA-Aa increased the levels of Sirt1 and catalase and the activation of ERK and Nrf2. Sirtuins are a group of 7 enzymes in which a"?> whose pathway has an important role in metabolic homeostasis and redox balance [70]. They are located not only in the nucleus but also in the cytoplasm, interacting with relevant cytoplasmic targets such as ERK [71]. ERK is a MAPK that has a crucial role in the maintenance of cellular homeostasis, acting in stress and proliferative responses [72] at least in part through Nrf2 activation [73]. Nrf2 is a crucial factor involved in oxidative stress protection mechanisms [74]. Given that, it is involved in the regulation of anti-inflammatory and cell survival genes such as phase II detoxifying/antioxidant enzymes as catalase [75]. Catalase is a key enzyme in the adaptive response against H_2_O_2_ [76]. Based on our results, we suggest that the pharmacological activation of Sirt1 is involved in oxidative stress response promoted by the EA-Aa, probably through the activation of the ERK/Nrf2 pathway which increases catalase-mediated antioxidant defense. Plants that have phenolic compounds as part of its constitution have already been represented as potent sirtuin activators [77–80] and consequently the pathways associated with it.

Altogether, our results showed the composition of typical plant extract molecules, but the different composition of EA-Aa allowed the lower toxicity compared to the others extracts and it is related to the antioxidant capacity we show. EA-Aa has a direct effect on the protection of proteins, DNA and lipids and presented the same effect in Cos-7 cells and in *C. elegans*, by the activation of the Sirt1/Nrf2 pathway. Thus, the low toxicity and the relevant potential in the maintenance of body's redox balance, allied to low cost and natural abundance, support further studies to explore the development of products based on it leaves to be used for both prevention and treatment of diseases related to oxidative stress.

## Figures and Tables

**Table 1:** Chemical composition identified from the LC-PDA of the extracts of *A. aculeata* leaves (mg · g^−1^ ± DP).

Retention time (min)	Peak	Compound	EA-Aa	EE-Aa	EM-Aa
2.31	1	Gallic acid	201.6 ± 1.4	159.4 ± 0.9	192.9 ± 1.1
4.91	2	Vanillic acid	182.4 ± 0.9	—	—
6.31	3	Caffeic acid	124.6 ± 1.2	119.7 ± 0.7	123.4 ± 1.0
8.83	4	Ferulic acid	197.9 ± 1.0	182.7 ± 1.0	189.6 ± 1.3
21.75	5	Rutin	74.8 ± 0.4	77.3 ± 0.2	75.9 ± 0.5
24.42	6	Quercetin	88.7 ± 0.2	87.6 ± 0.5	89.2 ± 0.3

**Table 2:** Chemical composition identified from the GC-MS of the extracts of *A. aculeata* leaves (mg · g^−1^ ± DP).

Retention time (min)	Peak	Compound	Molar mass	EE-Aa	EM-Aa
16.46	1	Campesterol	400	21.0 ± 0.11	18.9±−0.33
17.02	2	Stigmasterol	412	25.7 ± 0.22	34.7 ± 0.29
17.72	3	*β*-Sitosterol	414	60.1 ± 0.25	23.2 ± 0.27
18.89	4	Lupeol	426	49.4 ± 0.57	71.6 ± 0.64
21.01	5	Lupeol acetate	468	52.7 ± 0.63	55.1 ± 0.53

**Table 3:** Antioxidant activity of free radical scavenging DPPH and ABTS of EA-Aa, EE-Aa and EM-Aa (IC_50_*μ*g·mL^−1^).

	DPPH			ABTS		
	IC_50_	Maximum activity		IC_50_	Maximum activity	
		*μ*g·mL^−1^	%		*μ*g·mL^−1^	%
AA	2.68 ± 0.3	10	93.8	2.1 ± 0.2	10	98.8
EA-Aa	117.10 ± 7.3	250	82.2	47.4 ± 10.7	500	96.4
EE-Aa	12.92 ± 1.5	50	88.7	13.4 ± 0.4	100	99.8
EM-Aa	13.28 ± 1.2	100	90.8	10.5 ± 1.2	50	100

IC_50_: concentration required to capture 50% of the free radicals from the reaction.

## Data Availability

The datasets generated during and/or analyzed during the current study are available from the corresponding author upon reasonable request.

## Additional Points

*Highlights*. Antioxidant potential of the extracts was proved from *in vitro* to *in vivo* assays. The aqueous extract showed no toxicity, making its future therapeutic use possible. The aqueous extract activates the Sirt1/Nrf2 pathway and increases catalase levels. *A. aculeata* reduces oxidative damage and improves *C. elegans* stress resistance.
